# Immune Suppression in Tumors as a Surmountable Obstacle to Clinical Efficacy of Cancer Vaccines

**DOI:** 10.3390/cancers3032904

**Published:** 2011-07-18

**Authors:** Grégoire Wieërs, Nathalie Demotte, Danièle Godelaine, Pierre van der Bruggen

**Affiliations:** Ludwig Institute for Cancer Research and Université catholique de Louvain, de Duve Institute, 74 av. Hippocrate, P.O. Box B1-7403, B-1200 Brussels, Belgium; E-Mails: gregoire.wieers@bru.licr.org (G.W.); nathalie.demotte@bru.licr.org (N.D.); daniele.godelaine@bru.licr.org (D.G.)

**Keywords:** anergy, immunosuppression, cancer vaccines, galectin, tumor-infiltrating lymphocytes

## Abstract

Human tumors are usually not spontaneously eliminated by the immune system and therapeutic vaccination of cancer patients with defined antigens is followed by tumor regressions only in a small minority of the patients. The poor vaccination effectiveness could be explained by an immunosuppressive tumor microenvironment. Because T cells that infiltrate tumor metastases have an impaired ability to lyse target cells or to secrete cytokine, many researchers are trying to decipher the underlying immunosuppressive mechanisms. We will review these here, in particular those considered as potential therapeutic targets. A special attention will be given to galectins, a family of carbohydrate binding proteins. These lectins have often been implicated in inflammation and cancer and may be useful targets for the development of new anti-cancer therapies.

## 1. Introduction

Before the molecular identification of human tumor antigens, the existence of a spontaneous anti-tumor T cell response in melanoma patients was strongly suggested by the fact that CD8 cytolytic T cells (CTL), isolated from tumors and expanded for several weeks *in vitro*, lysed autologous melanoma cells. Moreover, some of these expanded tumor-infiltrating lymphocyte (TIL) populations were re-injected in patients together with IL-2 and produced objective tumor responses [1]. More recently, B. Dréno and coworkers have shown in a randomized trial that relapse-free survival of patients with only one invaded lymph node was extended if they received, in an adjuvant setting, adoptive transfer of autologous TIL previously expanded *in vitro* [2]. In the blood, the frequencies of CTL precursors that lyse autologous melanoma cell lines (anti-tumor) were evaluated by limiting dilution analysis and ranged from 1/1,000 to 1/100,000 of CD8 blood cells [3-5]. Frequencies of anti-tumor T lymphocytes isolated from subcutaneous melanoma metastases were ten times higher.

The molecular proof of the existence of human tumor-specific antigens came with the cloning of the first gene encoding a human melanoma antigen recognized by CTL: MAGE-1 [6]. The still ongoing search for additional antigens has yielded a long list of well-defined tumor antigens, many of which have been tested as targets for immunotherapy [7-9]. Various therapeutic vaccines have been injected in melanoma patients: peptides and proteins with or without adjuvants, viruses containing antigen-coding sequences, antigen-presenting cells loaded with antigens [10-18].

Most of these vaccines induced anti-vaccine T cells. The proportions of patients with detectable anti-vaccine T cells in their blood were highly variable. At first, no correlation was evidenced between immune T cell response to the vaccine and objective clinical response or tumor regressions [19-21]. Because CTL response to vaccinations was not readily detectable *ex vivo* in the blood, even in those patients who showed tumor regressions, sensitive and quantitative approaches were set up allowing the detection of frequencies of CTL precursors specific for a MAGE-3 peptide presented by HLA-A1 (anti-MAGE-3.A1) as low as one precursor per million CD8 blood T cells [22,23]. Notwithstanding their weakness, the observed anti-vaccine CTL responses were correlated with clinical evidence of tumor regression [15,18,23]: Among 20 melanoma patients, who received a vaccine aimed at inducing anti-MAGE-3.A1 CTL response and experienced tumor regression, 10 showed an anti-vaccine T cell response. This is in contrast with only one T cell responder among 30 vaccinated patients with progressive tumors.

In addition to T cell frequencies in the blood, other parameters can be informative to evaluate the response to the vaccine. In patients vaccinated with mature dendritic cells, pulsed with gp100 and tyrosinase peptides, delayed-type hypersensitivity (DTH) was observed in all the patients after a subcutaneous challenge. Anti-gp-100 or anti-tyrosinase CTL were derived only from DTH sites of those patients who experienced tumor regression [24]. Taken together, these observations suggest that vaccination can result in anti-vaccine T cell responses and that the induced T cells can both reach the tumor sites and trigger tumor destruction.

Nevertheless therapeutic vaccination of metastatic melanoma patients with these antigens is followed by tumor regressions of clinical significance in less than 10% of the patients. Even considering the absence of toxicity, the current vaccines are not effective enough to become generally applicable, as reviewed by different authors [18,19,25,26]. Our favorite hypothesis, which is shared by many tumor immunologists, to explain the lack of effectiveness of therapeutic vaccines observed in most patients is a local immunosuppression at the tumor sites. This hypothesis would also explain why melanoma patients usually mount a spontaneous T cell response against their tumor without being able to eradicate their tumor.

In this review, we will focus on the T lymphocytes that infiltrate tumors. Can we consider T cell infiltration as a clinical prognostic factor? Are these TIL functional or anergic? What are the mechanisms orchestrated by the tumor that would result in T cell dysfunction? We do believe that the tumor micro-environment inhibits by the same mechanisms both the spontaneously generated anti-tumor T cells and the anti-vaccine T cells. Our hope is to better understand the immune suppression mechanisms orchestrated by the tumor so as to be able to improve the clinical efficacy of cancer vaccines.

## 2. Does the Presence of TIL Correlate with Patient Survival?

### 2.1. In Melanoma

The correlation between patient survival and T cell-infiltrate in primary melanoma has been examined in about 20 papers published since the late seventies [27-47]. In Table 1, we have summarized the eight studies that included more than 180 patients.

In the first study, Larsen and Grude reported in 1978 a better overall survival of the patients with an intense lymphocytic infiltration “*completely surrounding the part of the melanoma that invaded the normal tissues*” [27]. Ten years later, Clark introduced his melanoma classification based on thickness and invasion that became a widely accepted prognostic model [31]. Clark also introduced the notion of brisk T cell infiltrate: “*TIL were present throughout the substance of the vertical growth phase or present and infiltrating across the entire base of the vertical growth phase*”. Since then, the general view was that a brisk T cell infiltration was a good clinical prognostic factor in melanoma.

In the nineties, the systematic biopsy of the sentinel lymph node (SLN) was introduced, which is the hypothetical first lymph node or group of nodes reached by metastasizing cancer cells from a primary melanoma [48]. Absence of TIL in the primary melanoma is predictive of the presence of metastatic tumor cells in the SLN [43,49]. The detection of metastasis in the SLN is a bad prognosis factor, decreasing 5-year survival from 89% (negative SLN) to 43% (positive SLN). In the latest multivariate analyses, which now include the SLN status, the presence of TIL in the primary melanoma is not an independent predictive factor for survival [43,45].

In melanoma metastases, higher numbers of TIL have been associated with a better survival [34,36,46]. Considering that most of the data were obtained with lymph node metastases, where it is difficult to distinguish between tumor-infiltrating lymphocytes and lymph node lymphocytes invaded by tumor cells, interpretation of these results requires caution.

### 2.2. In other Histological Types of Tumors

Beyond melanoma, there is growing evidence that dense TIL infiltrate is associated with a longer survival (Table 2).

In various histological types of tumors, the presence of TIL is unanimously considered as a good clinical prognostic factor: colon adenocarcinoma [50-59], ovarian carcinoma [60-63], endometrial carcinoma [64], non-small-cell lung carcinoma [65-67], lymphoma [68-71], urothelial carcinoma [72-74], esophageal carcinoma [75,76], hepatocellular carcinoma [77,78], oral squamous cell carcinoma [79-81], nasopharyngeal carcinoma [82-84], glioblastoma [85-87], breast carcinoma [88,89], and prostate carcinoma [90].

Colon adenocarcinomas without any sign of metastasis had more T cell infiltrates than tumors with pathological signs of early metastatic invasion such as vascular emboli, lymphatic invasion or perineural invasion [53].

Presence of TIL is not always of good prognosis, e.g., in squamous cell carcinoma [91] and renal cell carcinoma [92,93]. Considering that renal cell carcinoma patients were often treated by immunotherapy, *i.e.*, injections of IL-2 and IFN-α, the fact that tumor infiltration by T cells is of bad prognosis seems contra-intuitive, but considering that TIL numbers usually increase with the tumor grade, the simplest explanation for this correlation is that the grade of the tumors were not taken into account. However, the positive impact of immune cells on tumor growth cannot be excluded.

Noteworthy, TIL populations contain not only CD8 T cells able to kill tumor cells and CD4 T helper cells that secrete cytokines supporting proliferation of other T cells, but also regulatory T cells (Treg) able to inhibit function and proliferation of other T cells, and T cells that secrete, e.g., chemokines attracting macrophages and vasoendothelial growth factor (VEGF) promoting neoangiogenesis. In other words, TIL could also indirectly promote tumor progression [94].

## 3. What Is the Antigen Specificity of TIL?

It is tempting to think that the survival advantage conferred by the presence of TIL is due to the presence of tumor-antigen specific T cells in the tumor. However, T cell trafficking to the tumor site is not antigen-specific, as it is due to the recruitment of blood circulating T cells that express the appropriate adhesion molecules and chemokine receptors. What are therefore the evidences for anti-tumor T cell accumulation in the tumor mass?

### 3.1. Analysis of the TCR Diversity at the Tumor Site

T cell receptor (TCR) genomic loci undergo somatic recombination, plus the addition and removal of bases at recombination junctions, in order to generate the repertoire of structurally diverse TCR necessary for antigen recognition. The different TCR-β subunit chains, which have been estimated at 1 million per individual [95], can be unambiguously identified by their hypervariable “Complement Determining Region 3” sequence, which is the principal site of antigen contact. Upon antigen recognition, a T cell can proliferate and thus, by clonal amplification, the TCR-β transcript expressed by this T cell is multiplied. Evidence of focal TCR-β enrichment has been interpreted as the result of accumulation of T cells recognizing tumor antigens, within the tumor, despite the canonical view that tumor-specific T cells, which have encountered their cognate antigen, proliferate in a secondary lymphoid organ, and are subsequently recruited at the tumor site. Such enrichment was reported for melanoma [96-102], and for other tumors such as non-small-cell lung carcinomas, hepatocellular carcinomas, renal cell carcinomas, gliomas and oral squamous cell carcinomas [103-107]. Analysis of the TCR-γ transcripts was also used as an approach to evidence focal T cell enrichment [101].

Evidence for enrichment in one TCR transcript was followed for a few patients by molecular identification of the antigen recognized by the corresponding CTL, which was isolated either from the tumor or from the blood. This led to the identification of four MAGE-C2 peptides, one MAGE-6 peptide, one gp100 peptide and one peptide encoded by a mutated gene encoding myosin class I [108-112].

Clearly in favor of TIL proliferation *in situ* is the observation of tertiary lymphoid structures in some tumors. Tertiary lymphoid structures are lymph node-like structures inside the tumor mass composed of dendritic cells, T cells and B cells organized in germinal centers that could be sites of antigen presentation [113,114]. In non-small-cell lung cancer patients, the presence of tertiary lymphoid structure was even correlated with a better survival [114]. More information about the concept of tertiary lymphoid structures can be found in Pages *et al.* [113].

### 3.2. Study of TIL with HLA-Peptide Multimers

Study of the TCR diversity indicates accumulation of TCR clonotypes, suggesting the presence of anti-tumor T cells, but often without indication about the antigens recognized by these T cells. Hence the use of HLA-peptide multimers was introduced to identify some of the antigens targeted by TIL. We have only considered experiments with TIL that were not expanded before analysis. Using HLA-A2 multimers, which were folded with four peptides derived from melanoma differentiation proteins, the cumulative frequency of multimer-positive TIL ranged from 0 to >2% of the TIL (4/16 and 6/16 patients, respectively). Interestingly, these frequencies of anti-tumor T cells were close to the frequencies observed for anti-hepatitis CD8 T cells detected in liver biopsies of patients chronically infected with hepatitis C virus. Using a pool of HLA-A2 multimers, which were folded with four peptides derived from non-structural proteins of hepatitis C, frequencies of multimer-positive T cells ranged from 0 to >2% of the TIL (2/6 and 2/6 patients, respectively) [115]. In other reports, similar analyses were performed with TIL expanded for a few days *in vitro* [116-118]. In these reports, however, frequencies cannot be estimated.

### 3.3. Repertoire of Antigens Recognized by TIL before and after Immunotherapy

The frequencies of anti-vaccine T cells infiltrating metastases of a melanoma patient, who was vaccinated with MAGE-3.A1 and experienced tumor regression, were analyzed in details. These frequencies were compared with frequencies of TIL directed against tumor antigens other than the vaccine antigens (referred to as “anti-tumor TIL”). No anti-vaccine T cells were detected before vaccination in the blood or in the tumor. After vaccination, the frequency of anti-vaccine T cells was 1/67,000 of CD8 T cells in an invaded lymph node, 6-fold higher than in the blood [110]. After vaccination, anti-tumor TIL were about 10,000 times more frequent than anti-vaccine T cells inside metastases.

Taken together, these results suggest that the anti-vaccine CTL are clearly not the main effectors that kill the bulk of the tumor cells, but that their interaction with the tumor generates conditions enabling at least part of the numerous anti-tumor CTL to participate to the destruction of the tumor cells. Naive T cells appear to be stimulated in the course of this process as new anti-tumor clonotypes arise after vaccination. Indeed, one CTL clonotype, specific for a peptide encoded by a mutated caseinolytic protease, represented up to 7% of the TIL in metastases, whereas the corresponding TCR clonotype was not detected before vaccination [110,119,120]. Similar observations were made in another vaccinated patient [111].

An example of clonotype spreading—a diversification of the T cell clonotypes recognizing the same antigen—was also observed. An anti-MAGE-C2_336-344_.A2 T cell clonotype was detected in the blood and in the tumor before and after vaccination, but another anti-MAGE-C2_336-344_.A2, which recognized the same peptide/HLA was found at 1/11,000 in the blood only after vaccination and represented up to 9% of the CD8 TIL [110,120]. Clonotype spreading was also observed in a metastatic melanoma patient who experienced a complete clinical response after adoptive transfer of an anti-Melan-A.A2 CTL clone [121]. This clinical response correlated with an increased frequency of anti-Melan-A.A2 CTL in the blood, but the TCR sequence of this blood clonotype was different from the TCR sequence of the injected T cell clone.

## 4. Is Patient Survival Correlated with Signs of TIL Activity *in situ?*

### 4.1. Markers of TIL Activation and Proliferation

Expression of activation markers CD137 or CD25 on TIL was used as markers of TIL that have recently encountered their antigen. Expression of these markers in the peritumoral region was associated with a better survival [39,122]. Considering that regulatory T cells also express these receptors, the significance of the expression of these two markers is questionable.

To detect TIL proliferating at the tumor site, the presence of nuclear protein Ki-67 was used as a marker of cell division [123]. Higher numbers of TIL expressing Ki-67 were associated with a better survival in renal cell carcinoma and in hepatocellular carcinoma [78,93].

### 4.2. Co-Localization of TIL with Apoptotic Cells

The TUNEL method labels fragmented DNA, which is a characteristic of apoptotic cells. In seminoma, 2% of tumor cells were considered apoptotic by this method and half of them were in contact with at least one CD3 cell [124-126]. Co-localization of TIL with apoptotic cells was also examined in four gliomas. Two-thirds of the tumor cells that were in close contact with TIL, *i.e.*, which form immunologic synapses, were in apoptosis whereas only 25% of the tumor cells that were not in contact with TIL displayed signs of apoptosis [127].

### 4.3. Signs of Cytolytic Potential

The consensus that seems to emerge from studies on colon adenocarcinoma, hepatocellular carcinoma, melanoma and lymphoma, is that the presence of granzyme B^+^ CD8 TIL is a favorable prognosis factor [44,58,70,77]. In colon carcinoma, the frequency of granzyme B^+^ cells among the CD8 TIL can reach 30% [50]. This frequency is comparable to the 10% of granzyme B^+^ cells among the CD8 T cells in the liver of patients with hepatitis B, but it can reach 40% in hepatitis B patients that have an autoimmune disease characterized by T cell hyperreactivity [128-130].

Upon activation, CTL express CD107a at their surface for a few hours. This lysosomal-associated membrane protein (LAMP-1 or CD107a) has been described as a marker of degranulation of CD8 T cell upon stimulation. This marker was found at the surface of 1% to 9% of the TIL in colon adenocarcinoma metastases and seminoma [131-133]. Considering that no comparison was made between tumor tissues and non-cancerous tissues with an ongoing efficient cytolytic T cell response, one may wonder if this level of CD107a expression has to be considered as a sign of good or poor lytic capacity.

### 4.4. Production of Cytokines

The optimal cytokine pattern for an efficient anti-tumor immune response may differ in various tumor types. Cytokines have not been examined directly in the tumors but the subtypes of infiltrating cells have been studied by detection of specific transcription factors. The transcription factors that specify lineage commitment in T helper cells have started to be deciphered and the generally accepted model considers T-bet and GATA-3 as the master regulators of the so-called Th1 and Th2 differentiation, respectively, with c-Maf as the downstream factor that selectively controls IL-4 gene transcription [134,135]. TIL were analyzed for GATA-3 and T-bet expression by immunohistochemistry in 69 pancreatic tumor tissues. The median value of GATA-3/T-bet ratio was 5.2—this should promote a Th2 response. A 36-month survival analysis indicated a shorter survival by 15 months for patients with a GATA-3/T-bet ratio above the median value [136]. On the contrary, the 10-year survival of patients with Hodgkin lymphoma was significantly longer if the CD4 TIL infiltrate contains a higher frequency of c-Maf cells, which should produce IL-4 and be considered as Th2 [71].

TNF-α can be secreted by several types of tumor-infiltrating cells, e.g., fibroblasts, macrophages, NK cells, T cells, and could exert cytotoxic functions on tumor cells (reviewed in Bazzoni *et al.* [137]). TNF-α mRNA or protein was detected in non-small-cell lung cancers, hepatocellular carcinomas, colon adenocarcinomas or in the serum of gastric cancer patients, and was associated with a better survival [78, 138-140]. However, in terminal cancer patients, TNF-α is also implicated in cachexia, a loss of adipose and skeletal muscle mass, and accordingly elevated TNF-α in the serum of lymphoma and breast cancer patients was correlated with poor survival [141-145].

### 4.5. Concluding Remarks

Taken together, these results give the impression that TIL are functional at tumor sites. This is in contrast with the results of the T cell function assays described in the next section. One possible explanation is that most anti-tumor TIL are anergic and that only few functional TIL are detected by immunohistochemistry. Another explanation is that the functional TIL detected by immunohistochemistry have been activated outside the tumor, e.g., by antigens derived from Epstein-Barr virus or cytomegalovirus, and have been recently attracted into tumors by chemokines.

## 5. Are TIL Functional When Tested *ex vivo?*

Even the first sign of T cell activation, *i.e.*, calcium increase in the cytosol, was poor when TIL from numerous tumors were re-stimulated *ex vivo*, *i.e.*, tested on untreated and uncultured samples [146]. However, if TIL are isolated from the tumor microenvironment and expanded *in vitro* for days or weeks, they are usually able to secrete cytokines and kill tumor cells [147-149]. During this culture period, either functional TIL can be selectively amplified or dysfunctional TIL can recover their functions. The function recovery can be fast: after 48 h of culture without IL-2, up to 40% of the TIL isolated from three different tumors produced IFN-γ upon stimulation, whereas less than 10% produced IFN-γ when tested *ex vivo* [146]. We summarize below the functional tests performed *ex vivo* with human TIL.

### 5.1. Impaired Synapse Formation

T cells isolated from chronic lymphocytic leukemia patients showed impaired immunological synapse formation with leukemic B cells associated with defective actin polymerization [150]. Defective synapse formation between T cells and tumor B cells was also observed with cells isolated from patients with acute myeloid leukemia or follicular lymphoma [151,152].

### 5.2. Cytokine Secretion

Anti-melan-A^MART-1^ specific CD8 T cells were isolated with HLA-peptide multimers from melanoma or blood samples and short-term stimulated with peptide-pulsed cells. Eight percent of these TIL contained IFN-γ, whereas 44% of the multimer^+^ T cells isolated from blood did contain IFN-γ [148]. Anti-cytomegalovirus CD8 TIL were also isolated with HLA-peptide multimers, from the same tumors. Thirty percent of them contained IFN-γ upon stimulation with peptide-pulsed cells, whereas 38% of anti-cytomegalovirus blood CD8 T cells contained IFN-γ. In another study with one melanoma patient, polyclonal CD8 T cells isolated from ascites did not secrete IFN-γ upon short-term stimulation with tumor cells but were able to secrete IFN-γ upon stimulation with the cells pulsed with an EBV peptide [153].

How to explain the observations that anti-virus T cells were functional, whereas the anti-tumor cells were not? If the tumor environment is suppressive, why were the anti-virus T cells not sensitive to suppression? One explanation is that anti-virus T cells were residing in the tumor only for a short time. Another explanation is that anti-tumor T cells have been repetitively stimulated by tumor antigens and are therefore more sensitive to immunosuppression.

We have tested CD8 and CD4 TIL, which were isolated from melanoma and carcinomatous ascites of the ovary, colon, and pancreas. TIL were stimulated with beads coated with anti-CD3 and anti-CD28 antibodies, which is a strong stimulus. Compared to donor blood T cells, CD8 and CD4 TIL secreted low levels of IFN-γ and TNF-α [154].

### 5.3. Lytic Activity

CD8 TIL isolated from renal cell carcinoma, malignant pleural effusions, ovarian and pancreatic carcinoma ascites failed to lyse cells coated with anti-CD3 antibodies in an assay known as a redirected killing assay, whereas CD8 blood T cells were cytolytic [154,155]. Presence of perforin was used in several studies as a marker for the cytolytic potential of TIL [148,156], but one could also argue that TIL that have very recently lysed tumor cells have lost most of their perforin.

### 5.4. Concluding Remarks

Taken together, the few results reported above give the impression that most TIL are dysfunctional in *ex vivo* functional assays whereas the reports on signs of TIL activity *in situ* gave the opposite impression. Because the *ex vivo* functional assays are rather straightforward, we are tempted to conclude that most TIL are actually dysfunctional. We hope that additional experiments from different groups will help strengthen this conclusion. There is also a need for functional assays with control T cells isolated from non-cancerous tissues such as acute inflammatory sites.

## 6. Mechanisms of Immune Suppression in Tumors as Potential Therapeutic Targets

A reasonable hypothesis is that the tumor microenvironment impairs TIL functions. We will focus in this part of the review on the various mechanisms able to decrease TIL efficacy, in particular the mechanisms that could be inhibited by pharmacological methods. We will not discuss the selection of tumor cell variants that no longer present the antigens that are the targets of TIL. This tumor escape mechanism has been described in detail [157-159].

### 6.1. Inhibition by Surface Receptors

Several molecules expressed by TIL could down-modulate their activation upon antigen recognition. A more complete set of such molecules is described in Table 3, together with potential drugs tested *in vitro* or in clinical trials.

#### 6.1.1. Competing for a Positive Costimulus

CTLA-4, expressed on T cells, can compete with CD28 for the binding to CD80/86, expressed on antigen-presenting cells and, consequently, prevents recruitment of PKCθ at the synapse and T cell activation [160]. Addition of an anti-CTLA-4 blocking antibody to a co-culture of anti-CD3-stimulated human T cells and CD80-expressing cells increased the secretion of IL-4, IL-5 and IFN-γ [161]. In mice, the engagement of CTLA-4 resulted in decreased TCR signaling, decreased IL-2 transcription, and cell cycle arrest [162]. However, this has not been demonstrated with human T cells. TIL were shown by flow cytometry to express CTLA-4 in Hodgkin disease, melanoma and ovarian carcinoma [163-165]. CTLA-4^+^ TIL isolated from melanoma that also expressed Programmed Death-1 (PD1) produced very low levels of IFN-γ upon stimulation with PMA-Ionomycin, a strong non-specific stimulus [163].

Anti-CTLA-4-blocking monoclonal antibodies Ipilimumab and Tremelimumab have already been tested in many clinical trials, both in melanoma patients and in patients with other malignancies [166-175] (see also http://www.clinicaltrials.gov/). In a phase-3 study, 676 unresectable stage III or IV melanoma patients received either a vaccine containing two gp100 peptides corresponding to antigens expressed by melanoma and normal melanocytes, or Ipilimumab alone, or Ipilimumab plus the gp100 vaccine [166]. The median overall survival was 10.1 months among patients receiving Ipilimumab plus gp100 and 10 months among patients receiving Ipilimumab alone, as compared with 6.4 months among patients receiving gp100 alone (hazard ratio for death, 0.68; P < 0.001). Adverse events can be severe, long-lasting, or both, but most are reversible with appropriate treatment [166,176]. Ipilimumab was approved by the U.S. Food and Drug Administration in March 2011 for treatment of metastatic melanoma patients. Further studies on the exact role of CTLA-4 are clearly needed. James Allison, who discovered CTLA-4, recently wrote: Understanding the precise mechanism of CTLA-4 activity *in vivo*, and by extension, the mechanism of anti-tumor immune activity mediated by CTLA-4 blockade, is an area of active investigation [177].

#### 6.1.2. Bringing Phosphatases Close to the TCR Complex

Several receptors, e.g., PD1 and Killer Inhibitory Receptors (KIR), are phosphorylated upon ligand binding and, consequently, recruit SHP-1 [178-182]. It is generally assumed that bringing SHP-1, a phosphatase, close to the cytosolic parts of the TCR complex alters the phosphorylation cascade that follows antigen recognition [183]. *Ex vivo* treatment of TIL with sodium stibogluconate, a drug known to inhibit SHP-1 and SHP-2, restored their ability to flux calcium upon TCR triggering with superantigens but did not restore their ability to secrete IFN-γ [146]. This drug is used for treating patients with Leishmaniasis. It has not been used yet for cancer patients, perhaps because of its side-effects.

Blockade of PD-1/PDL-1 interactions seems to prolong survival of T cells and promote their expansion rather than reverse T cell dysfunction. Addition of blocking anti-PD-1 antibodies in cultures of human T cells stimulated with peptide-pulsed dendritic cells increased by 2–3 fold the proliferation of anti-peptide T cells [184], whereas addition of blocking anti-PD-L1 antibodies in cultures of human T cells which were stimulated with PD-L1^+^ tumor cells or peptide-pulsed blood mononuclear cells, either diminished aptotosis of specific T cells [185], or increased their proliferation by 2–3 fold [186]. Anti-PD1 antibodies were administered in two phase I clinical studies. In both trials the antibodies were well tolerated. Humanized antibody CT-011 was injected in 17 patients with hematologic malignancies, among whom one had a complete remission [187]. Another anti-PD1 antibody, MDX-1106, was injected in 39 patients with solid tumors [188]. One durable complete response and two partial responses were seen. Tumor biopsies from nine patients undergoing treatment were analyzed by immunochemistry for PD-L1 expression. Among the four patients with a PD-L1-positive staining, three experienced tumor regressions, whereas no regression was observed among the five patients with a PD-L1-negative biopsy.

There is some rationale for combining therapies with different antibodies as blood CD8 T cells from cancer patients can co-express multiple inhibitor receptors, such as PD-1 and Tim-3 [189]. Upon antigen stimulation, these T cells proliferated slightly more if both anti-PD-L1 and anti-Tim-3 blocking antibodies were added, compared to cultures containing only one of the two antibodies. Increased proliferation was similarly observed with PD1^+^ CTLA-4^+^ CD8 T cells, which were isolated from the liver of hepatitis C-infected patients and stimulated with peptide in the presence of anti-PD-L1 and anti-CTLA-4 blocking antibodies [190].

#### 6.1.3. Cautionary Remarks

The mere detection of inhibitory receptors on TIL, is sometimes considered as a marker of TIL impairment [191-193], despite the fact that many of these receptors are also expressed by recently activated T lymphocytes with normal functions. A better argument in favor of a local inhibition at the tumor site would be to show by immunohistochemistry the presence of both the inhibitory receptor on TIL and the ligand on tumor or stroma cells.

### 6.2. Immunosuppressive Cells

Numerous immune cell types have some level of immunosuppressive activity. Among them are Treg, myeloid-derived suppressor cells, mesenchymal stem cells and tumor-associated macrophages. Only the presence of Treg and macrophages has been associated with survival prognosis and will be discussed below.

#### 6.2.1. Regulatory T Cells

CD4^+^ T cells with a CD25^+^FOXP3^+^ phenotype are generally considered as Treg. In humans, this definition includes regulatory T cells but also activated CD4^+^ T cells [219, 220]. Other cell surface markers were proposed to define human Treg, but none of these markers is specifically expressed by Treg because they can also be found on activated T cells (reviewed by Sakaguchi [221]). Treg could inhibit T cell functions by different mechanisms that have been reviewed by Shevach [222].

The lack of specificity of these markers can explain the contradictory reports on Treg and survival. The relevance of most of these studies is therefore questionable. Infiltration by CD4^+^CD25^+^ or CD4^+^FOXP3^+^ was correlated with poor survival in renal cell carcinomas, gastric carcinomas, breast carcinomas, ovarian carcinomas, non-small-cell lung carcinomas, melanomas and colon adenocarcinomas [65,223-232]*,* but the presence of Treg was correlated with a greater survival in nasopharyngeal carcinoma, gastric carcinoma and lymphoma patients [71,80,84,233,234].

The most specific marker of human Treg up to now is an epigenetic marker: the demethylation of the first intron of *FOXP3* [219,220,235]. However, it has not yet been used to examine if the presence of Treg in human tumors is a prognostic factor for survival.

Different treatments aimed at reducing the number of CD25^+^ regulatory T cells were administered to cancer patients (see www.clinicaltrials.gov): cyclophosphamide, anti-CD25 monoclonal antibody Daclizumab, and Denileukin diftitox (Ontak^®^), an engineered protein combining IL-2 and diphtheria toxin [236-238]. This protein can bind to CD25 and introduce the diphtheria toxin into cells that express those receptors, killing the cells.

Cyclophosphamide was injected i.v. at low doses or given *per os* [239-241]. A two-fold depletion of CD4^+^CD25^+^ T cells was documented only after *per os* administration. It was also injected i.v. at high doses and induced lymphodepletion, therefore possibly removing a cytokine sink [242,243]. Melanoma patients were injected with a high dose of cyclophosphamide, which led to lymphodepletion, in combination with total body irradiation, adoptive T cell transfer and IL-2 infusions [244]. Eighteen of the 25 patients who have received this combined treatment experienced tumor rejection but also severe side effects.

Daclizumab was efficient in depleting transiently CD4^+^CD25^+^ T cells. However, Treg are only a subset of CD4^+^CD25^+^ T cells. When the demethylation of FOXP3 intron 1 was used to detect Treg frequencies, neither Daclizumab nor Denileukin diftitox was able to halve the frequency of Treg cells [238]. Administration of Daclizumab had no significant effect on the progression-free survival of melanoma patients vaccinated with mature dendritic cells pulsed with tumor peptide and keyhole limpet hemocyanin [245]. Furthermore, it appeared to blunt the anti-vaccine T cell response, as anti-vaccine CD8 T cells were not detected in any of the Daclizumab-treated patients [245]. This effect is probably a consequence of the transient expression of CD25 on the surface of activated T cells.

#### 6.2.2. Tumor-Associated Macrophages

Infiltration of tumor by macrophages was correlated in several malignancies with an increased tumor angiogenesis and poor prognosis [246-254]. Activated macrophages can secrete TGF-β and activate latent TGF-β into active TGF-β [255]. Macrophages can also express amino-acid depleting enzymes IDO, arginase and NO synthase, and harbor cell surface inhibitory receptors such as PD-L1 and B7-H4 [256-259].

Macrophages and cancer cells can also express the enzyme cyclooxygenase 2 (COX2) which metabolizes arachidonic acid into prostaglandin E2 (PGE2) [260-262]. PGE2 was shown to inhibit *in vitro* human T cell proliferation and production of IFN-γ and IL-2, while it spared the production of Th2 cytokine IL-4 [263-265]. Thus, these observations assimilated PGE2 to an immunosuppressive molecule. High expression of COX2 in cancers was reported to be associated with a shorter survival [260-262]. Noteworthy, COX2 was measured in these studies but not PGE2. Non-steroidal anti-inflammatory drugs, which are COX inhibitors, were developed to inhibit prostaglandin production. In mice, COX inhibitors inhibited the prostaglandin pathway and reduced arginase production by MDSC [266]. Drugs commonly used in the clinic could thus reduce arginine depletion at the tumor site. Sildenafil, a phosphodiesterase-5 inhibitor frequently used as a vasodilatator, was shown to down-regulate arginase and NO synthase and restore *in vitro* proliferation of T cells isolated from patients with multiple myeloma or head and neck cancer [266,267].

Tumor-associated macrophages were associated with a favorable prognosis in cutaneaous melanoma [40,268,269]. Could it be that macrophages have cytotoxic properties that are exerted within the tumor? Is it because macrophages are accompanied by other immune cells, including T cells, which could be considered as a good prognostic factor?

### 6.3. Soluble Inhibitory Molecules

We describe below soluble molecules that can modulate TIL activation and function. A more complete set of molecules is shown in Table 4, together with potential drugs tested in clinical trials or *in vitro*.

#### 6.3.1. Cytokines

*In vitro*, the active forms of TGF-β and IL-10 are classically described as immunosuppressive [270-274]. However, because both cytokines have pleiotropic indirect roles, it is difficult to evaluate how they impact on TIL function. They are nevertheless considered as important therapeutic targets. Three phase I clinical trials with the anti-TGF-β antibody GC1008 are ongoing in patients with melanoma, renal cell carcinoma, and relapsing malignant mesothelioma (see www.clinicaltrials.gov). No clinical results have yet been reported. Fresolimumab, another anti-TGF-β blocking antibody, has been tested in systemic sclerosis, but not in cancer patients [275].

#### 6.3.2. Amino-acid deprivation

Indoleamine Dioxygenase (IDO) is an enzyme that catalyzes the first step in tryptophan catabolism along the kynurenine pathway. Breakdown of tryptophan inside IDO-expressing tumor cells results in the consumption of available tryptophane in the local tumor environment and thus in deprivation of this essential amino-acid for the T cells [285]. The metabolites generated by IDO, such as L-kynurenine, can induce T cell hyporesponsiveness and death, and render dendritic cells immunosuppressive [299-301]. Stimulation of human T cells with anti-CD3 antibodies in the presence of tryptophan metabolites impeded T cell proliferation [299].

It has been proposed that tryptophan starvation and presence of kynurenines can convert murine naive conventional CD4 T cells into highly suppressive regulatory CD4 T cells. In turn, regulatory T cells would induce IDO expression in dendritic cells, which can further expand the regulatory T cell compartment [302]. After 6 days of co-culture of IDO^+^ mature dendritic cells with autologous human T cells, frequencies of CD4^+^CD25^+^Foxp3^+^CD127^-^ T cells increased by more than 2-fold. This was prevented by the addition of 1-methyl tryptophan to the co-culture [303].

IDO is overexpressed in a variety of tumors and is upregulated in many cells in response to IFN-γ [285].

In metastatic melanoma patients, the patients with a short survival (mean: 8 months) had all an IDO-expressing tumor, as detected by immunochemistry, whereas only 20% of the long survivors (mean: 262 months) had an IDO-positive tumor [304]. IDO expression was reported to be a negative prognostic factor of survival in ovarian carcinoma and colon adenocarcinoma [305-307], and a good prognosis factor in hepatocellular carcinoma and basal-like breast carcinoma [308,309]. The presence of IDO can be considered both as the sign of a hostile environment for T cells and as a marker of an ongoing immune response with IFN-γ-secreting T cells. It is therefore not surprising that correlations between survival and the mere marker IDO lead to apparently paradoxical results.

Two other enzymes that degrade amino-acids were described to play an immunosuppressive role in cancer: arginase and NO-synthase [258,310]. Both enzymes metabolize arginine, an essential amino-acid for T cells.

#### 6.3.3. Tumor Metabolites

The metabolism of tumor cells can generate high amounts of lactic acid, due to up-regulation of glycolytic enzymes and hypoxia at the tumor site [311]. Adding lactic acid to culture medium was shown to block cytokine production and diminish cytotoxic activity by 50% and, therefore, TIL function could be inhibited by this tumor metabolite [312,313].

By hydrolyzing ATP, hypoxic tumor cells could release adenosine [314]. Adenosine could also be produced in the tumor microenvironment by ectonucleotidases CD39 and CD73 that are expressed at the surface of some lymphoma cells and normal leucocytes [296,315,316]. Concentrations of adenosine in adenocarcinomas were 10- to 20-fold higher than in normal tissue [317], and adenosine was shown to inhibit *in vitro* cytokine production and cytotoxic activity by CD4^+^ and CD8 T cells [295].

#### 6.3.4. Reactive Oxygen Species

Peroxynitrites, which belong to reactive oxygen species, seem to be produced at the tumor site in human prostate carcinoma and in different human carcinoma cell lines [310,318]. In mice, reactive oxygen species have been shown to be secreted by myeloid-derived suppressor cells [310,319], and peroxynitrites have been shown to induce nitration of murine TCR-CD8 complexes, resulting in altered antigen recognition [283,319].

In humans, monocytes and a subpopulation of neutrophils, described as myeloid derived suppressor cells, were shown to produce reactive oxygen species [320]. A subpopulation of granulocytes, also described as myeloid derived suppressor cells, were shown to produce arginase-I and, under limiting amounts of L-arginine, reactive oxygen species may be transformed into peroxynitrites [321]. Exposure of human blood T cells to peroxynitrites down-regulated their activation upon stimulation [322].

#### 6.3.5. Glycoprotein/Galectin-3 Lattice

We have observed that human CD8 TIL, in contrast with CD8 blood cells, show impaired IFN-γ secretion upon *ex vivo* re-stimulation. We have attributed the decreased IFN-γ secretion to a reduced mobility of T cell receptors upon trapping in a lattice of glycoproteins clustered by extracellular galectin-3 [323]. Indeed, we have shown that TIL harbor surface galectin-3 and that treating TIL with N-acetyllactosamine (LacNAc), a galectin ligand, restored this secretion. We have also shown the same phenomenon with CD4 TIL. We will further examine the roles of galectins in the next chapter.

## 7. Are Galectins Targets for Improving the Clinical Efficacy of Cancer Vaccines?

### 7.1. Introduction

Galectins belong to a family of lectins, *i.e.*, sugar- or glycan-binding proteins. Galectins share the ability to bind β-galactosides and contain homologous carbohydrate recognition domains (CRD). Their binding affinity for simple β-galactosides, such as disaccharides or trisaccharides, is relatively weak. However, galectin binding to natural glycoconjugate ligands expressed on cell surfaces or in the extracellular matrix is usually of higher affinity, in the submicromolar range. We will discuss below the role in the tumor microenvironment of the sole galectin-1, -3, and -9. Further reading on galectins can be found in reviews by Rabinovich [324] and Cummings [325].

Galectins have been classified in three structural groups: (a) the prototype galectin-1 with a single CRD, which can form homodimers via non-covalent interactions; (b) the chimeric galectin-3 with a single CRD and a large amino-terminal domain, which contributes to self-aggregation into pentamers; and (c) the tandem-repeat galectins with two different CRD and a 5 to 70 amino-acid linker, e.g., galectin-9.

Galectins are found in the cytosol and in the nucleus where they interact with proteins to regulate growth, cycle progression and apoptosis [326,327]. Galectins lack a signal peptide but can nevertheless be secreted by an unknown mechanism. Extracellularly, the different roles of extracellular galectins are related to their ability to bind sugar moieties on surface *O*- and *N*-glycoproteins. The interactions of galectins and glycoproteins are complex and cannot be interpreted in terms of interactions between a ligand and a receptor. Several factors contribute to high-affinity binding to natural glycoconjugate ligands: the natural multivalency, the oligomeric state of the galectins and the multivalency of their natural glycoconjugate ligands [325].

Galectins participate in both glycoprotein clustering and glycoprotein/galectin lattices, and could therefore modify receptor signaling and increase the surface half-life of some glycoproteins [328]. In tumors, galectin-1, -3, -9 are often secreted by tumor cells and monocyte-derived cells, but can also be secreted by activated B or T cells [329-335]. The inhibition of T cell proliferation by CD4^+^CD25^high^ cells, possibly Treg cells, was reported to be reversed by an anti-galectin-1 antibody [336]. T cell proliferation was also inhibited in the presence of mesenchymal stromal cells, which secreted high levels of galectin-1 and galectin-3. T cell proliferation was restored by adding thiodigalactoside, a ligand competitor of both galectins, or by inhibiting galectin-3 expression in mesenchymal stromal cells by RNA interference [337-340].

### 7.2. Galectins as a Prognosis Factor?

Sera of melanoma and colon adenocarcinoma patients were reported to contain more galectin-3 than sera of healthy volunteers [341,342]. Melanoma patients with more than 10 ng/mL were reported to have a median survival of less than 5 months, whereas the median survival of patients with less than 8 ng/mL was 60 months [343]. No correlation was found, however, between tumor regression in melanoma patients vaccinated with tumor antigens and concentration of galectin-3 [344]. In head and neck squamous cell carcinoma patients, elevated galectin-3 and galectin-1 in serum were also correlated with a worse survival [345]. Is the presence of galectins a sign of immune suppression and better conditions for metastasis or the mere sign of an advanced disease and high tumor burden?

### 7.3. Galectins Diminish T Cell Function and Viability

Blood T cells, T cell lines or Jurkat T cells were stimulated in the presence of galectin-1, -3, or -9, usually added in the culture at μM concentrations. Treated cells either entered in apoptosis [346-349], proliferated less or secreted more IL-10 [350]. It was also reported that a Hodgkin lymphoma cell line, which expressed galectin-1, had a negative effect on proliferation of CD4 T cells [351,352] and that this effect diminished when the lymphoma cells knocked down for galectin-1 were added to the co-culture. T cell apoptosis mediated by galectins could be a physiological mechanism taking place in the thymus during early development of T cells, as thymic epithelial cells can secrete galectin-1 and *in vitro* exposure to galectin-1 induced apoptosis of subsets of CD4^lo^ CD8^lo^ thymocytes [353].

Galectin-1, -3 and -9 added at μM concentrations also induce apoptosis of prostate carcinoma, melanoma, B-lymphoma and leukemia cells, provided they harbor the adequate glycosylation pattern [346,354-357]. We did not observe apoptosis of blood T cells, T cell clones or tumor cell lines when galectin-3 was added at 10 nM, which is the highest concentration of galectin-3 that we measured in carcinoma ascites. It is nevertheless possible that galectins could accumulate in solid tumors in confined microenvironments and reach concentrations in the μM range.

The apoptosis induced by galectin-1 and galectin-3 seems to be mediated by their interactions with highly glycosylated surface molecules, such as CD43 and CD45, as these molecules co-purified with these galectins and were associated in patches at the T cell surface [329,358-360]. Modification of the glycosylation pattern of T cells rendered them resistant to galectin-1-induced apoptosis [361-363]. The apoptosis induced by galectin-9 requires its binding to glycan moieties on Tim-3, a negative costimulatory receptor expressed on some activated T cells [189,298].

We have reported that treatment of TIL with galectin ligand LacNAc boosted their secretion of cytokines upon stimulation. Why do galectin-3 ligands improve TIL function? Our working hypothesis is that TIL have been stimulated by antigen recently, and that the resulting activation of T cells could modify the expression of enzymes of the N-glycosylation pathway and change the structure of *N*-glycans exposed at the cell surface, as shown for murine T cells [364]. We surmise that the recently activated TIL, compared to resting T cells, harbor a set of glycans that are either more numerous or better ligands for galectin-3. Galectin-3 is abundant in many solid tumors and carcinomatous ascites, and can thus bind to surface glycoproteins of TIL and form lattices that would thereby reduce mobility of surface receptors implicated in T cell activation. This could explain the impaired function of TIL. The release of galectin-3 by soluble competitor ligands would restore mobility of surface receptors and boost IFN-γ secretion by TIL. We recently strengthened this hypothesis by showing that CD8 TIL treated with an anti-galectin-3 antibody had an increased IFN-γ secretion [154].

*Ex vivo* treatment of TIL with galectin-3 ligand LacNAC boosted their functions [323]. We have thus searched for galectin-3 ligands available for clinical use. GCS-100^®^, a modified citrus pectin with cytotoxic properties on tumors, was administered by i.v. injection to 24 multiple myeloma patients [365-368]. Side effects were minor. *In vitro*, GCS-100 detached galectin-3 from TIL and boosted cytotoxicity and secretion of different cytokines [154]. Pectasol^®^, another modified citrus pectin, was given orally to 10 prostate cancer patients [369]. Seven of them experienced an increased doubling time of blood PSA, which suggests a decreased tumor growth.

DAVANAT^®^, a galactomannan derived from guar gum, has been shown to bind to galectin-1 [277], and to increase the anti-tumor activity of chemotherapy drug 5-fluorouracil in mice. It was injected together with 5-fluorouracil in 25 patients with solid tumors without major side effects [276]. Short *ex vivo* treatment of TIL with DAVANAT boosted secretion of cytokines and lytic activity (our own unpublished data). We intend to launch a clinical trial with metastatic melanoma patients where MAGE3.A1 and Na17.A2 peptides, which correspond to tumor-specific antigens expressed by various types of tumor, will be administered together with systemic and local injections of DAVANAT.

## 8. Concluding Remarks

There is now an increasing number of available data on human tumors and TIL to support the scenario of an immunosuppressive tumor microenvironment. The respective contribution of the different immunosuppressive mechanisms remains to be clarified in order to define whether different lymphocyte populations are impaired through different mechanisms, and whether several inhibitory pathways add up in the same cells. Considering the remarkably low toxicity of therapeutic vaccination of cancer patients, provided the target antigen is tumor-specific, every possible effort to improve its efficacy should be done. It was proposed that, in some vaccinated patients, a few anti-vaccine T cells reach metastases, succeed in reversing the immunosuppression, and trigger a broad activation of other anti-tumor T cells that proceed to eliminate the bulk of the tumor cells [18,110,111,120]. In other words, the anti-vaccine T cells serve only as a “spark” that activates the regression of the tumor. This hypothesis is supported by gene expression profiling of tumor samples resected before vaccination. The gene signatures that are associated with clinical benefit to vaccines reflect an immune response in the tumor present prior to vaccinations [370-373].

In the absence of vaccinations, treatments aimed at boosting TIL functions in vaccinated patients could also trigger activation of anti-tumor T cells. The same treatments should also contribute to provide a better tumor microenvironment for the anti-vaccine T cells able to reach the tumor sites.

We should keep in mind that boosting TIL function may result in increased secretion of molecules that indirectly promote tumor progression and that simultaneously blocking several surface inhibitory receptors may increase the severity of side-effects.

## Figures and Tables

**Table 1.** Patient survival and presence of tumor-infiltrating lymphocyte (TIL) in primary melanoma.

	**Nb of patients** a	**T cell infiltration** b	**5-year survival**		**Sentinel Lymp Node (SLN)** d

			%	Correlation with T cell infiltrate c	
Larsen, 1978 [27]	669	TIL+	78		
		TIL+++	91	+	-
Johnson, 1985 [29]	262	TIL+	48		
		TIL+++	60	+	-
Clark, 1989 [31]	386	TIL absent	59 e		
		TIL brisk	89		
		TIL non-brisk	75	+	-
Clemente, 1996 [35]	285	TIL absent	37		
		TIL brisk	77		
		TIL non-brisk	53	+	+^f^
Tuthill, 2002 [38]	259	TIL absent	71		
		TIL brisk	100		
		TIL non-brisk	71	+	-
Taylor, 2007 [43]	887	TIL absent	75		
		TIL brisk + non-brisk	76	-	+
Mandalà, 2009 [45]	1251	TIL absent	90		
		TIL brisk + non-brisk	95	+ g	+
Barnhill, 1996 [33]	650	TIL absent	86		
		TIL present	90	-	-

a Only studies including >180 patients were considered;

b Number of TIL estimated by immunohistology;

c Log-rank. “+” means significant;

d Sentinel Lymph Node (SLN) biopsy procedure included in the analysis;

e Proportion of patients with 8-year overall survival;

f SLN^+^ excluded from the analysis;

g Not confirmed by the multivariate Cox proportional hazard model.

**Table 2.** Patient survival correlates with the presence of TIL in various tumor types.

	**Nb of patients** a	**T cell infiltration**	**5-year survival (%)**	
**Colon adenocarcinoma**	276	absent	30	*
Ropponen, 1997 [59]		weak	45	
		mild	70	
		dense	75	
**Colon adenocarcinoma**	371	CD8^+^ low b	50	*
Chiba, 2004 [52]		CD8^+^ high	80	
**Colon adenocarcinoma**	959	CD45RO^+^ <250/mm^2^	24	
Pagès, 2005 [53]		CD45RO^+^ >250/mm^2^	46	
**Colon adenocarcinoma**	286	CD3^+^ low b	85	* c
Laghi, 2009 [56]		CD3^+^ high	100	
**Hepatocellular carcinoma**	302	CD8^+^ GrB low b	45	
Gao, 2007 [77]		CD8^+^ GrB high	60	
**Lung neoplasms**^d^	1290	CD8^+^ <20% of all cells	30	d
Ruffini, 2009 [67]		CD8^+^ >20% of all cells	40	
**Non-small-cell lung cancer**	219	absent	36	e
Kilic, 2009 [66]		present	76	
**Endometrial carcinoma**	368	CD8^+^ <4/0.3 mm^2^	60	* e
de Jong, 2009 [64]		CD8^+^ >4/0.3 mm^2^	85	
**Prostate carcinoma**	325	rare/absent f	30	* e
Vesalainen, 1994 [90]		moderate	55	
		dense	70	
**Renal cell carcinoma**	221	CD8^+^ scanty f	85	
Nakano, 2001 [93]		CD8^+^ aboundant	60	
**Ovary Carcinoma**	186	CD3^+^ absent	5	
Zhang, 2003 [60]		CD3^+^ present	38	
**Breast Cancer**	1334	CD8^+^ absent	50	* g
Mahmoud, 2011 [89]		CD8^+^ present	65	

* Estimated on survival curves.

a Only studies including >180 patients were considered.

b High *versus* low TIL = above or below the median value.

c Patients with lymph node metastasis were excluded.

d Correlation between TIL infiltrate and survival observed for adenocarcinoma and squamous cell carcinoma.

e 10-year survival analysis.

f Number of TIL estimated by immunohistochemistry.

g 20-year survival analysis.

**Table 3.** Surface inhibitory receptors expressed by TIL and counteracting strategies.

**Inhibitory receptor**		**Ligand**	**Could be reversed by**	**Function tested**	
***Reversion tested in a clinical trial***					

**CTLA-4**^a^	CD152	CD80	anti-CTLA-4 antibody	proliferation	[161,166-173,175,194,195]
		CD86		cytokine secretion	
				lytic activity	
**PD1**^a^	CD279	PD-L1 / -L2	anti-PD1 antibody	proliferation^b^	[146,184-188,196-198]
			SHP-1 inhibitor Stibogluconate		
***Reversion tested in vitro—drug used for other purposes***					

**KIR2DL1**^c^	CD158a	HLA Class I	Valproïc acid treatment of the tumor cells	lytic activity	[199-201]
**KIR2DL2/3**	CD158b				
**KIR3DL1**	CD158e				
**CEACAM1**^a^, ^c^	CD66a	CEACAM1	vitamin D		[202-204]
***Reversion tested in vitro—no drug available yet***					

**BTLA**^a^, ^b^	CD272	HVEM	anti-BTLA antibody	proliferation	[191,205,206]
**BY55**	CD160		anti-HVEM antibody	cytokine secretion	
**Tim-3**^a^			anti-Tim-3 antibody	proliferation	[189,207-210]
				lytic activity	
				cytokine secretion	
**NKG2A** a	CD159a	HLA-E	anti-CD94 or anti-NKG2A antibodies	lytic activity	[211-213]
**KLRD1**	CD94		anti-TGFβ or anti-rIL-15α antibodies		
**ILT2**	CD85j	HLA-G	anti-ILT2 antibody	proliferation	[214,215]
				lytic activity	
**NKRP1A**^c^	CD161	LLT1	anti-NKRP1A antibody	cytokine secretion	[216]
**Ly-1**	CD5	CD72 / gp150	no compound described		[217,218]

a The inhibitory receptor is expressed upon T cell activation.

b A vaccination protocol was recently proposed in order to avoid expression of inhibitory receptor BTLA on anti-vaccine T cells [191].

c This receptor could also have positive co-stimulus properties.

**Table 4.** Soluble molecules with a suppressive activity on TIL and counteracting strategies.

**Inhibitory molecule**	**Could be reversed by**	**Function tested**	
***Reversion tested in a clinical trial***			

**TGF-β**	anti-TGF-β antibody		CT01112293^a^
***Reversion tested in vitro—drug used for other purposes***			

**Galectin-1**	galactomannan DAVANAT®	proliferation	[276,277]
		cytokine secretion	
**Galectin-3**	modified citrus pectin GCS-100®	cytokine secretion	[154] + unpublished data
	galactomannan DAVANAT®	lytic activity	
**PGE2^b^**	COX inhibitors	TCR signaling	[265,278-281]
		proliferation	
		cytokine secretion	
***Reversion tested in vitro—no drug available yet***			

**Arginase / NO synthas**	NOHA	proliferation	[282-284]
	1-NMMA		
**IDO**	dextro-1-methyl tryptophane	proliferation	[285-289]
	IDO inhibitors		
**IL-10**	anti-IL-10 antibody	lytic activity	[273,274,290-294]
	Anti rIL-10 antibody	proliferation	
		cytokine secretion	
**Adenosine**	CD39 inhibitor ARL67156	lytic activity	[295-297]
	methylxanthines: caffeine and theophylline	cytokine secretion	
**Galectin-9**	no compound described		[189,298]

a Described in http://www.clinicaltrials.gov.

b Can also induce secretion of pro-inflammatory cytokines.
